# Evaluation of immunoserological detection of anti-liver kidney microsomal, anti-soluble liver antigen and anti-mitochondrial antibodies

**DOI:** 10.1038/s41598-023-37095-z

**Published:** 2023-06-20

**Authors:** Alejandro Campos-Murguia, Nicole Henjes, Stephanie Loges, Heiner Wedemeyer, Elmar Jaeckel, Richard Taubert, Bastian Engel

**Affiliations:** 1 Department of Gastroenterology, Hepatology, Infectious Diseases and Endocrinology, Hannover Medical School, Carl-Neuberg-Straße 1, 30625 Hannover, Germany; 2 Member of the European Reference Network for Hepatological Diseases (ERN RARE-LIVER), Hamburg, Germany; 3 Present Address: Ajmera Transplant Center, Toronto General Hospital, United Health Network, University of Toronto, Toronto, Canada

**Keywords:** Primary biliary cirrhosis, Diagnostic markers, Autoimmune diseases

## Abstract

Autoantibodies are the diagnostic hallmark of autoimmune liver diseases. Indirect immunofluorescence (IFT) is the reference method for the detection of anti-mitochondrial antibodies (AMA) and anti-liver kidney microsomal type-1 (anti-LKM1) antibodies, and inhibition ELISA (iELISA) for anti-soluble liver antigen (anti-SLA) antibodies. Given the complexity of these techniques, commercial ELISAs have emerged as a practical alternative, but without head-to-head validations. This study evaluated the agreement between three commercial ELISAs and the reference techniques and the impact of polyreactive immunoglobulin G (pIgG), a recently described phenomenon in autoimmune hepatitis, on commercial ELISAs. Inter-rater reliability was assessed using Cohen-Kappa coefficient (κ). Forty-eight, 46, and 66 samples were analyzed for AMA, anti-LKM1, and anti-SLA, respectively. For AMA, one commercial assay showed high agreement (κ = 0.91 (0.78–1.00)) with the reference method, while the other two showed weak or moderate agreement. For anti-LKM1, only one commercial assay showed high agreement (κ = 0.86 (0.71–1.0)). For anti-SLA antibodies only moderate agreement was achieved (κ up to 0.71 (0.52–0.89)). There was a trend towards higher pIgG levels in false-positives in the commercial ELISAs. Patients with high suspicion of autoimmune liver diseases should be referred to reference laboratories with the capacity of performing gold standard methods if the initial ELISA-based screening was performed.

## Introduction

Autoantibody testing is the most important screening method for detecting an autoimmune origin in the work-up of any hepatitis. The diagnosis of autoimmune liver diseases (AILD) is clinically challenging and requires the combination of biochemical, serological, imaging, and, in the case of autoimmune hepatitis (AIH), histological features, as pathognomonic features are usually absent. In primary biliary cholangitis (PBC), anti-mitochondrial antibodies (AMA) constitute the conventional autoantibody for the diagnosis, while anti-liver kidney microsomal type-1 antibodies (anti-LKM1), and anti-soluble liver antigen antibodies (anti-SLA), are part of the conventional repertoire for the diagnosis of AIH and relevant for subclassification^1,2^.

Screening for autoantibodies can be performed with immunofluorescence testing (IFT) and various enzyme-linked immunosorbent assays (ELISAs). While IFT on three rodent tissue sections can detect multiple autoantibodies at once, e.g., antinuclear antibodies (ANAs), anti-smooth muscle antibodies (anti-SMAs), anti-LKM1, AMA, and anti-liver cytosol antibodies type 1 (anti-LC1), antigen-specificity is typically proven by ELISAs, which also can be used as a primary screening approach^3^. Additionally, IFT is insensitive for anti-SLA, the autoantibody with the highest specificity for AIH, which was first detected by competitive inhibition ELISA (iELISA)^4,5^. IFT and iELISA are complex techniques that require highly qualified personnel and are time-consuming, leading to the development of commercial ELISAs as a convenient alternative.

Whereas the current EASL guidelines endorse the detection of ANA, anti-SMA, anti-LKM, and AMA on rodent tissue sections using IFT^2^, the AASLD guidelines do not elaborate on a specific technique^1^. Nevertheless, IFT is considered the gold standard for antibody detection^5^, however, discordance between IFT and ELISA has recently been demonstrated for ANA detection^6^ and also for the detection of anti-LKM1 depending on the manufacturer of the commercially available ELISA^7^. Hence, it is important to perform external validation of these commercial assays, which are often tested in healthy individuals as a comparator prior to their approval, to avoid misdiagnosis and delays in initiating appropriate treatment.

Recently, the presence of polyreactive immunoglobulin G (pIgG) was reported as a novel marker for the diagnostic workup of AIH with higher overall accuracy than the conventional ANA and anti-SMA and additional diagnostic value in seronegative AIH. PIgG binds to a variety of protein and non-protein blocking reagents commonly used as blocking reagents in ELISAs, which may lead to undesirable cross-reactivity that ultimately degrades diagnostic accuracy^8^.

This study aimed primarily to evaluate the agreement between three currently available commercial ELISA assays against IFT and an in-house iELISA for the detection of AMA, anti-LKM1, and anti-SLA. The second aim was to assess whether the presence of high pIgG levels could be associated with false positive (FP) results in commercial anti-LKM1 and anti-SLA assays.

## Material and methods

### Study population and sample selection

In this single center study, samples from our institutional biobank were evaluated. All samples with positive AMA (No. = 16) and anti-LKM1 (No. = 23) based on IFT, and anti-SLA (No. = 22) based on the in-house iELISA^4^ and complete clinical data were included and compared with controls with chronic liver disease, negative antibodies and complete clinical data. Patients with replicative viral hepatitis were excluded from the control group. Samples were matched by age at a ratio of 1:2 in the case of anti-SLA and AMA and 1:1 in the case of anti-LKM because the number of patients was insufficient for matching. Matching by age was decided since there is a well-known influence of age on autoantibody positivity, also aiming to reduce any potential selection bias of control samples^9^.

Diagnosis of PBC and AIH was based on current guidelines^1,2,10^. Because the purpose of this project was to evaluate inter-assay agreement, the definition of cases and controls was based on the presence or absence of antibodies using the conventional reference methods, i.e. IFT and the in-house iELISA. The cutoffs used were based on the current guidelines for the IFT and on local standardized cutoffs for the in-house iELISA^2,10,11^. For anti-LKM an IFT cut-off of 1:40 for adults and 1:10 in the case of children^2,11^, and for anti-LKM1 an iELISA cut-off of 40% were employed^12^. For AMA an IFT cut-off of 1:40^10^ or an iELISA cut-off of 40% were used^12^. Finally, in the case of anti-SLA, an iELISA cut-off 40% was applied^12,13^.

In addition, ALT, AST, and AP were obtained from the patients’ medical records at the time of sample collection, as were IgG levels in the case of anti-LKM and anti-SLA samples for its association with AIH and IgM levels in the case of AMA for its association with PBC.

The local Ethics Committee (protocol numbers 5582 and 2817-2015, MHH Ethikkommission, Hannover, Germany) approved this study. Written informed consent was obtained from all subjects in advance. All experiments were performed in accordance with relevant guidelines and regulations.

### Evaluation of antibodies by in-house competitive iELISA assays

In-house iELISA was performed as published^4,14^. Briefly, antibodies from defined anti-SLA/anti-LKM1 or AMA-positive indicator sera were coated overnight in a volume per well of 50 µl at room temperature in microtiter plates (Dynatech, el Paso, Texas for anti-LKM1, and Maxisorp, Nunc, Denmark for AMA and SLA). The supernatants were removed and after a washing step respective antigens were added. For the generation of the antigens rat liver was homogenized and centrifuged for 15 min at 3000 rpm. The pellet was discarded and the supernatant was centrifuged at 8500 rpm for 15 min. The mitochondrial fraction was collected from the pellet and used as antigen in the respective iELISA. The supernatant was further centrifuged for 60 min at 50,000 rpm, antigens for LKM were collected from the pellet and antigens for SLA were collected from the supernatant and were added to the respective iELISA. All antigens were added at a concentration of 100 µg/ml and incubated for 1 h at room temperature. Patient samples were diluted 1:10 in PBS + 10 mM EDTA and added to the microtiter plates following two washing steps. Incubation was done for 1 h at room temperature. Following three additional washing steps, avidin-peroxidase and sodium perborate dissolved in citrate buffer were added to microtiter plates. The photometric reaction was stopped after 5 min, and the absorbance was measured. The percentage of inhibition of the indicator serum to its respective autoantigen was used as a surrogate for the antibody titer. An extended description of the technique is available in the supplementary data.

### Antibody assessment by IFT

IFT was performed by experienced technicians using the recommended methodology of the guidelines issued in 2004 by the Committee for Autoimmune Serology of the International Autoimmune Hepatitis Group^3^. Briefly, a commercial rodent multi-organ substrate panel (kidney, liver and stomach) was used (LKS Rat wrapped Standard Kit, Aesku.Diagnostics GmbG & Co. Wendelsheim, Germany). The sera were diluted, starting with a dilution of 1:20 up to 1:160, and applied to the slide to cover the entire tissue section and allow binding of the autoantibodies to the substrates. After washing, the sample was exposed to a second fluorochrome-labeled antibody. Finally, once washed again, the slides were examined under fluorescence microscope (Olympus BX60 Microscope, Evident Europe GmbH, Germany), and the antibody staining pattern was evaluated and interpreted accordingly to the guidelines^3^.

### Antibody evaluation by commercial ELISA assays

All three antibodies were assessed with three different commercial assays according to the manufacturer’s instructions (Aesku.Diagnostics GmbG & Co. Wendelsheim, Germany; Euroimmun Medizinische Labordiagnostika AG, Lübeck; Germany; Inova Diagnostics, San Diego, California). All assays detect immunoglobulin G (IgG) autoantibodies. In the case of AMA, the three commercial assays detect antibodies against the antigenic E2 subunits of pyruvate dehydrogenase (PDH-E2), the 2-oxo-glutarate dehydrogenase complex (OGDH-E2) and the branched-chain 2-oxo-acid dehydrogenase (BCOADH-E2) (Aeskulisa AMA-M2-G, ref: 3705; Euroimmun Anti-M2-3-ELISA, ref: EA 1622-9601 G; Inova Diagnostics QUANTA Lite® M2 EP (MIT3) ELISA, ref: 704540). For anti-LKM1 the assays measure IgG against recombinant cytochrome p450 IID6 (CYP2D6) (Aeskulisa LKM-1, ref: 3703; Euroimmun LKM-1, ref: EA 1622-9601 G; Inova Diagnostics QUANTA Lite® LKM-1, ref: 708745). Finally, for anti-SLA, all the commercial kits use recombinant SLA (Aeskulisa SLA/LP, ref: 3704; Euroimmun SLA/LP, ref: EA 1302-9601 G; Inova Diagnostics QUANTA Lite® SLA, ref: 708775).

### Measurement of polyreactive immunoglobulin G (pIgG)

Measurement of pIgG was performed as originally described^8^. Plates (Maxisorp, Nunc, Denmark) were coated with huntingtin-interacting protein 1 related protein (HIP1R) fragment (MGQLQDQQALRHMQASLVRTPLQGILQLGQELKPKSLDVRQE) (Peps4LS GmbH, Heidelberg, Germany) and blocked with Tris-buffered saline (TBS) and bovine serum albumin (BSA) (Sigma-Aldrich, Germany). The plates were incubated with diluted sera (1:100; v/v) and then a secondary rabbit anti-human IgG labeled with horseradish peroxidase (Jackson ImmunoResearch Europe Ltd; Philadelphia, Pennsylvania) diluted in TBS was applied. The color reaction was performed with 3, 3′, 5, 5′ tetramethyl benzidine according to the manufacturer`s instructions (BioLegend, San Diego California), and the optical density was measured in an ELISA reader (Tecan Sunrise-Basic, Grödig, Austria) at 450 nm. Sera from five reference samples were used to construct a standard curve using previously defined (20/40/60/80/100) arbitrary units (AU)^8^.

### Statistical analysis

All statistical analysis was performed using R Statistical Software (version 4.1.2, R Core Team). The distribution of variables for demographic characteristics was assessed using the Shapiro–Wilk test. The matching procedure was performed using propensity score matching with the nearest available neighbor, without replacement or caliper, using the MatchIt package^15^. Because most numerical variables had a non-normal distribution, all variables are presented in median and range, while categorical variables are presented in frequencies and percentages. For comparisons between groups, the Mann–Whitney U test was used for continuous variables and the Chi^2^ test for categorical variables. For comparisons of non-parametric continuous variables between more than two independent groups, the Kruskal–Wallis test was performed, and the Dunn post-hoc test was used for multiple comparisons. A p-value of < 0.05 was considered statistically significant.

The results of the three commercial assays were compared using contingency tables with the IFT and in-house iELISA, and analyzed using the Cohen-kappa coefficient (κ) to measure inter-rater reliability using the Psych package^16^. To simplify the interpretation of the κ coefficient, a κ of ≤ 0.60 was interpreted as inadequate or low agreement, a κ between 0.61 and 0.79 as a moderate agreement, and a κ ≥ 0.80 as a high agreement^17^.

Alternative cutoffs for each commercial ELISA assay were computed using IFT as standard for AMA, and iELISA for anti-SLA and anti-LKM1, defining the optimal cut-off as the point maximizing the Youden function. The Cutpointr package was used to perform the receiver operator characteristic curve (ROC curve) and Youden Index analysis^18^.

### Ethics approval statement

This study was approved by the local Ethics Committee (protocol numbers 5582 and 2817-2015, MHH Ethikkommission, Hannover, Germany). Written informed consent was obtained from all subjects in advance.

## Results

### Anti-mitochondrial antibodies (AMA)

Forty-eight samples were included for the AMA analysis, 16 AMA positive samples based on IFT matched by age to 32 control samples of patients with chronic liver diseases and negative AMA. Demographic characteristics are shown in Table 1a. When evaluated by the commercial ELISAs, 9/48 (18.8%) samples tested positive with Aeskulisa (cut-off: 18.1 U/ml), 21/48 (43.8%) samples tested positive with Euroimmun (cut-off: 20 RU/ml), and 18/48 (37.5%) tested positive with Inova Diagnostics (cut-off: 25 U).

**Table 1:** General characteristics of the patients.

(a)		Reference range	AMA IFT negative (n = 32)	AMA IFT positive (n = 16)	p-value
	Age (years)		44.0 [38.0, 53.0]	30.0 [22.5, 37.5]	0.38
	Female		14 (60.9)	21 (91.3)	< 0.01
	Male		9 (39.1)	2 (8.7)	
	PBC diagnosis		0 (0.0)	15 (93.8)	< 0.01
	ALT (U/L)	≤ 34	47.0 [32.5, 85.5]	34.00 [26.5, 41.0]	0.12
	AST (U/L)	≤ 31	35.0 [26.5, 54.5]	31.00 [25.0, 45.0]	0.76
	AP (U/L)	≤ 104	162.0 [84.0, 281.5]	134.00 [73.0, 208.5]	0.37
	IgM (g/L)	≤ 2.3	1.1 [0.8, 1.4]	3.57 [2.3, 4.7]	< 0.01
	AMA iELISA positivity		2 (6.2)	16 (100.0)	< 0.01

(b)			Anti-LKM IFT negative (n = 24)	Anti-LKM IFT positive (n = 24)	
	Age (years)		44.00 [38.0, 53.0]	30.00 [22.5, 37.5]	0.01
	Female		14 (60.9)	21 (91.3)	0.04
	Male		9 (39.1)	2 (8.7)	
	AIH diagnosis		15 (65.2)	20 (87.0)	0.17
	ALT (U/L)	≤ 34	97.00 [42.5, 504.0]	51.0 [18.0, 116.5]	0.04
	AST (U/L)	≤ 31	93.00 [48.0, 454.0]	37.0 [23.5, 73.0]	0.02
	AP (U/L)	≤ 104	156.5 [126.5, 196.8]	114.0 [97.0, 144.0]	0.03
	IgG (g/L)	≤ 16	18.65 [12.0, 22.6]	12.5 [10.6, 15.1]	0.07
	anti-LKM1 iELISA positivity		1 (4.3)	14 (60.9)	< 0.01

(c)			Anti-SLA iELISA negative (n = 44)	Anti-SLA iELISA positive (n = 22)	
	Age (years)		53.0 [40.0, 66.0]	53.0 [29.8, 65.8]	0.63
	Female		27 (61.4)	15 (68.2)	0.79
	Male		17 (38.6)	7 (31.8)	
	AIH diagnosis		25 (56.8)	21 (95.5)	< 0.01
	ALT (U/L)	≤ 34	82.5 [32.5, 386.5]	46.0 [32.0, 166.0]	0.34
	AST (U/L)	≤ 31	100.5 [47.3, 396.8]	68.0 [31.0, 131.0]	0.10
	AP (U/L)	≤ 104	142.5 [90.5, 182.3]	97.5 [76.0, 131.3]	0.06
	IgG (g/L)	≤ 16	18.1 [11.0, 24.0]	18.1 [17.1, 21.9]	0.53

Patients’ characteristics of the patients’ samples tested for AMA (a), anti-LKM (b) and anti-SLA (c). Groups are stratified as positive and negative dependent on their antibody positivity as assessed by the respective reference method.

ALT, alanine transaminase; AIH, autoimmune hepatitis; AMA, anti-mitochondrial antibodies; Anti-LKM1, anti-liver kidney microsomal type 1 antibodies; Anti-SLA, anti-soluble liver antigen antibodies; AP, alkaline phosphatase; iELISA, inhibition enzyme-linked immunosorbent assay; IFT, indirect immunofluorescent test; IgG, immunoglobulin G; IgM immunoglobulin M; PBC, primary biliary cholangitis.

Data is provided as absolute number (frequency in %) or median [IQR].

**Figure 1:**
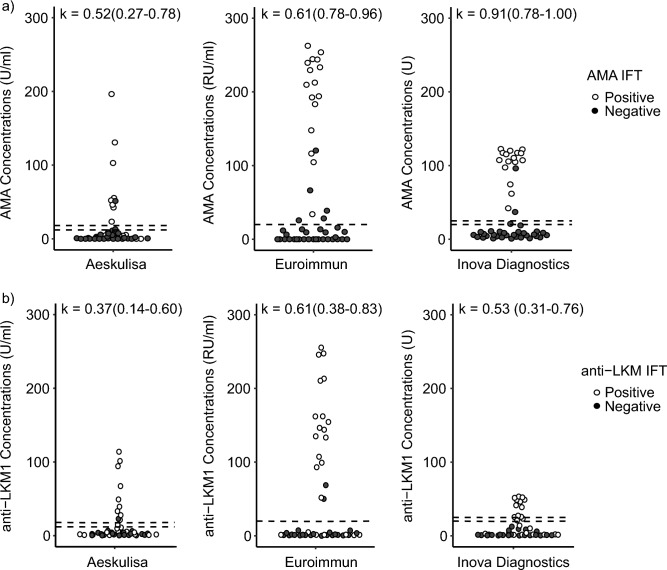
Classification of samples according to the commercial ELISAs antibody concentrations and the IFT (**a**), AMA; (**b**), anti-LKM). Concentrations given in U/ml. The dashed line represents the cut-off for each commercial assay (Aeskulisa: 18.1 U/ml, Euroimmun: 20 RU/ml and, Inova Diagnostics: 25 U). Aeskulisa (12–18 U/ml) and Inova Diagnostics (20.1–24.9 U) can have indeterminate results, indicated by the lower dashed lines. The color of the dots represents the classification according to the IFT. Cohen-Kappa coefficient with confident boundaries between Commercial ELISAs and IFT.

Agreement between commercial ELISA assays and the IFT as reference for AMA detection was heterogeneous, with Inova Diagnostics showing high agreement (κ = 0.91 (0.78–1.00)), Euroimmun showing moderate agreement (κ = 0.61 (0.78–0.96)) and Aeskulisa showing low agreement (κ = 0.52 (0.27–0.78)) (Fig. 1a). The low correlation with the Aeskulisa assay came at the expense of high false negative (FN) results (No. = 8), whereas Inova Diagnostics and Euroimmun did not show FN, with IFT as the reference (Table 2a).

**Table 2:** Performance of commercial ELISA based on IFT as reference for the detection of AMA and anti-LKM1.

		TP	FN	FP	TN
(a)	AMA				
	Aeskulisa	8	8	1	30
	Euroimmun	16	0	5	27
	Inova diagnostics	16	0	5	27
(b)	Anti-LKM1				
	Aeskulisa	9	13	1	22
	Euroimmun	16	7	2	21
	Inova diagnostics	11	10	0	23

Performance characteristics of different ELISA manufacturers in comparison to IFT as reference method for AMA (a) and anti-LKM1 (b).

AMA, anti-mitochondrial antibodies; Anti-LKM1, anti-liver kidney microsomal type 1 antibodies; FN, false negative; FP, false positive; TN, true negative; TP, true positive.

Comments: Aeskulisa (range: 12–18 U/ml) and Inova Diagnostics (range: 20.1–24.9 U) can have indeterminate results, this values were not included for this analysis.

**Figure 2:**
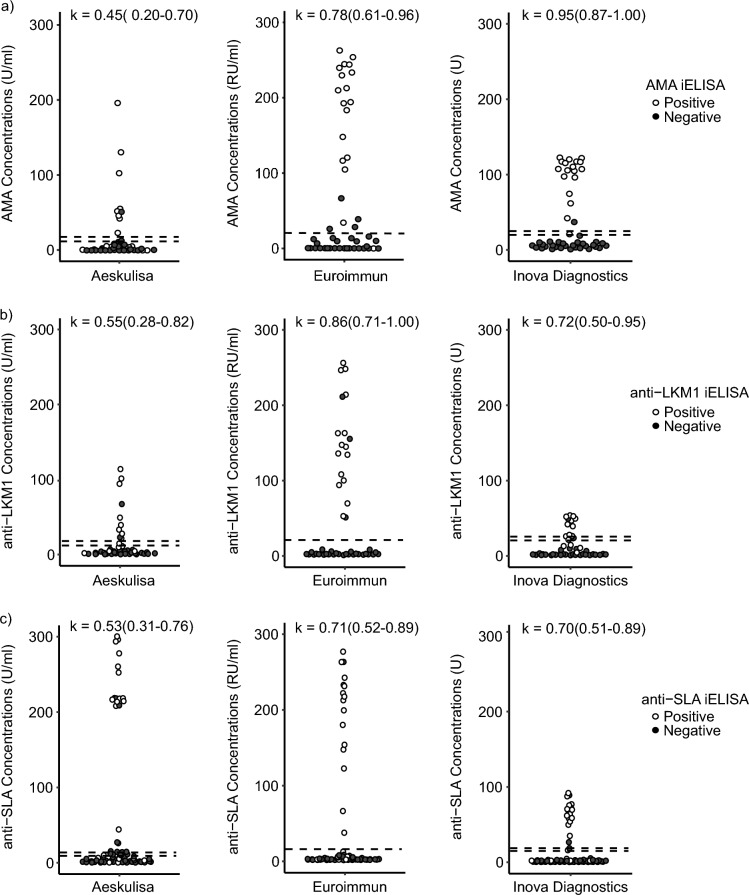
Classification of samples according to the commercial ELISAs and the in-house iELISA (**a**), AMA; (**b**), anti-LKM1; (**c**), anti-SLA. Concentrations given in U/ml. The dashed line represents the cut-off for each commercial assay (Aeskulisa: 18.1 U/ml, Euroimmun: 20 RU/ml and, Inova Diagnostics: 25 U). Aeskulisa (12–18 U/ml) and Inova Diagnostics (20.1–24.9 U) can have indeterminate results, indicated by the lower dashed lines. The color of the dots represents the classification according to the iELISA. Cohen-Kappa coefficient with confident boundaries between Commercial ELISAs and iELISA.

We also evaluated the agreement between the commercial ELISAs and the in-house AMA iELISA. Eighteen (37.5%) samples were positive for AMA as determined by the in-house iELISA. Again, Inova Diagnostics showed very high agreement (κ = 0.95 (0.87–1.00)), Euroimmun moderate agreement (κ = 0.78 (0.61–0.96)), and Aeskulisa low agreement (κ = 0.45 (0.20–0.70)) (Fig. 2a). As in the case of IFT, the low correlation from Aeskulisa came at the expense of high FN results (No. = 10) (Table 3a).

**Table 3:** Performance of commercial ELISA based on iELISA as reference for the detection of AMA, anti-LKM1, and anti-SLA.

		TP	FN	FP	TN
(a)	AMA				
	Aeskulisa	8	10	1	28
	Euroimmun	17	1	4	26
	Inova diagnostics	17	0	1	29
(b)	Anti-LKM1				
	Aeskulisa	8	6	2	29
	Euroimmun	15	0	3	28
	Inova diagnostics	10	4	1	19
(c)	Anti-SLA				
	Aeskulisa	15	6	7	33
	Euroimmun	15	7	1	43
	Inova diagnostics	15	6	2	41

Performance characteristics of different ELISA manufacturers in comparison to iELISA as reference method for AMA (a), anti-LKM1 (b) and anti-SLA (c).

AMA, anti-mitochondrial antibodies; Anti-LKM1, anti-liver kidney microsomal type 1 antibodies; Anti-SLA, anti-soluble liver antigen antibodies; FN, false negative; FP, false positive; TN, true negative; TP, true positive.

Comments: Aeskulisa (range: 12–18 U/ml) and Inova Diagnostics (range: 20.1–24.9 U) can have indeterminate results, which were not included in the analysis.

There was high agreement between the in-house iELISA and the IFT (κ = 0.91 (0.79–1.00)). The in-house iELISA showed no FN results and almost no FP results (No. = 2), with the IFT serving as the standard.

For the three commercial assays, alternative local cut-off values were calculated using an AUROC and Youden index to adjust the cut-off values to the local background, using the IFT as reference, as recommended by the ELISA manufacturers and previous publications^6^. These local cut-off values improved the κ-coefficient for Euroimmun (alternative cut-off: 69.2 RU/ml; κ = 0.91 (0.78–1.00)) and Inova Diagnostics (alternative cut-off: 42.0 U; κ = 0.95 (0.86–1.00)), but not for Aeskulisa (alternative cut-off: 2.7 U/ml, κ = 0.50 (0.27–0.73)) (Table 4a). ROC curves are shown in supplementary Fig. 1a.

**Table 4:** Original and alternative cut-off values.

		Original cut-off	Original κ	Alternative cut-off	New κ
(a)	AMA^a^				
	Aeskulisa	18.1 [U/ml]	0.52 (0.27–0.78)	2.7 [U/ml]	0.50 (0.27–0.73)
	Euroimmun	20 [RU/ml]	0.61 (0.78–0.96)	69.2 [RU/ml]	0.91 (0.78–1.00)
	Inova diagnostics	25 [U]	0.91 (0.78–1.00)	42.0 [U]	0.95 (0.86–1.00)
(b)	Anti-LKM1^b^				
	Aeskulisa	18.1 [U/ml]	0.55 (0.28–0.82)	9.6 [U/ml]	0.69 (0.47–0.92)
	Euroimmun	20 [RU/ml]	0.86 (0.71–1.00)	51.7 [RU/ml]	0.90 (0.78–1.00)
	Inova diagnostics	25 [U]	0.72 (0.50–0.95)	10.0 [U]	0.85 (0.69–1.00)
(c)	Anti-SLA^b^				
	Aeskulisa	18.1 [U/ml]	0.53(0.31–0.76)	34.6 [U/ml]	0.64 (0.44–0.84)
	Euroimmun	20 [RU/ml]	0.71(0.52–0.89)	11.1 [RU/ml]	0.75 (0.57–0.92)
	Inova diagnostics	25 [U]	0.70 (0.51–0.89)	3.4 [U]	0.61 (0.41–0.81)

Reporting of Original and optimized cut-off values. Cohen Kappa correlation coefficients are shown with corresponding 95% confidence intervals for AMA (a), anti-LKM1 (b) and anti-SLA (c).

^a^IFT as reference.

^b^In house iELISA as reference.

In summary, the AMA commercial assays show a heterogeneous correlation with the IFT and the in-house AMA iELISA. Inova Diagnostics shows good agreement, Euroimmun moderate agreement, and Aeskulisa low agreement with both IFT and the in-house iELISA. The low agreement of Aeskulisa came at the cost of a higher risk of FN results, limiting its use for screening purposes.

### Anti-liver/kidney microsome antibodies type 1 (anti-LKM1)

Forty-six samples were evaluated for the anti-LKM/anti-LKM1 analysis, 23 samples of patients with positive anti-LKM as evaluated by the IFT were compared with 23 control patients. Even though a matching by age was performed, an adequate balance was not achieved. However, matching guaranteed non-observer-biased selection of control samples. Four children (< 18 years) were included in this analyses, in whom a corresponding cut-off for IFT (1:10) was applied as published^11^. Additional demographic data are shown in Table 1b. As estimated by commercial ELISAs, 10/46 (21.7%) samples tested positive with Aeskulisa (cut-off: 18.1 U/ml), 18/46 (39.1%) samples tested positive with Euroimmun (cut-off: 20 RU/ml), and 11/46 (23.9%) tested positive with Inova Diagnostics (cut-off: 25 U/ml). None of the commercial assays for quantification of anti-LKM1 showed high agreement with the IFT for quantification of anti-LKM. Euroimmun showed the best but only moderate agreement (κ = 0.61 (0.38–0.83)), Aeskulisa showed the lowest agreement (κ = 0.37 (0.14–0.60)), and Inova Diagnostics also had low agreement (κ = 0.53 (0.31–0.76)) (Fig. 1b). For all three commercial anti-LKM1 assays, moderate or low agreement came at the expense of high FN results for anti-LKM (Table 2b).

Fifteen (32.6%) samples were positive by the in-house anti-LKM1 iELISA (cut-off: 40%). The agreement between the in-house anti-LKM1 iELISA and the commercial assays was high in the case of Euroimmun (κ = 0.86 (0.71–1.00)) (Fig. 2b). Inova Diagnostics showed moderate agreement (κ = 0.72 (0.50–0.95)) and Aeskulisa showed low agreement (κ of 0.55 (0.28–0.82)) (Fig. 2b), both again at the expense of high FN results (Table 3b).

There was moderate agreement between the in-house iELISA for quantification of anti-LKM-1 and the IFT for quantification of anti-LKM (κ = 0.57 (0.34–0.79)). The in-house iELISA showed high FN (No. = 9) and almost no FP (No. = 1) results, with the IFT serving as the standard.

Alternative local cut-off values were derived from the Youden Index, using the in-house iELISA as a reference because the IFT also detects other non-LKM1 antibodies. The results are presented in Table 4b and show slightly better κ-coefficients for all three assays than those with the original cut-off values. ROC curves are shown in supplementary Fig. 1b.

**Figure 3:**
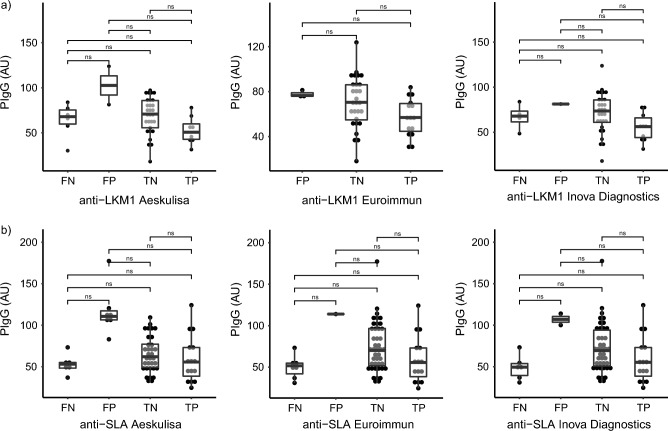
Correlation of polyreactive IgG with false positive results of commercial ELISAs. (**a**), anti-LKM1; (**b**), anti-SLA. Polyreactive IgGs (PIgG) values in Arbitrary Units (AU) in relation to the commercial ELISAs classified as False Negative (FN), False Positive (FP), True Negative (TN), True Positive (TP) with the in-house iELISA as reference. Statistical significance was evaluated with the Dunn’s post-hoc Test.

Finally, we classified the results of the commercial ELISAs as FP, FN, true negative (TN), and true positive (TP), using the in-house iELISA as a standard. We did not use the IFT as standard because it also detects anti-LKM other than type 1. We compared pIgG levels between these groups. Although a tendency toward higher pIgG values in FP was noted in the Aeskulisa results was noted, the number of samples with FP results (No. = 2) was not sufficient to show statistical significance (Fig. 3a).

In summary, commercial ELISAs for the detection of anti-LKM1 show only low or moderate agreement with the IFT. Correlation with the in-house anti-LKM1 iELISA was high in the case of Euroimmun, moderate in the case of Inova Diagnostics, and inadequate in the case of Aeskulisa. PIgG interference could not be proven in the measurement of anti-LKM1 with commercial assays.

### Soluble liver antigen antibodies (anti-SLA)

Twenty-two samples with positive anti-SLA as evaluated by the in-house iELISA (cut-off: ≥ 40%) were available for analysis, these samples were matched by age with 44 controls. (Table 1c). When evaluated with the commercial ELISAs, 22/66 (33.3%) samples were positive with Aeskulisa (cut-off: 18.1 U/ml), 16/66 (24.2%) samples were positive with Euroimmun (cut-off: 20 RU/ml) and 17/66 (25.8%) were positive with Inova Diagnostics (cut-off: 25 U). Agreement between the commercial assays and the in-house anti-SLA iELISA was moderate in the case of Inova Diagnostics (κ = 0.70 (0.51–0.89)) and Euroimmun (0.71 (0.52–0.89)), and low in the case of Aeskulisa (κ = 0.53 (0.31–0.76)) (Fig. 2c). In all cases at the expense of a high FN results (Table 3c).

Local cut-off values with the in-house anti-SLA iELISA as reference were calculated for the three commercial assays, achieving a slight improvement in the agreement of the Aeskulisa and Euroimmun assays, without reaching a high agreement (Table 4c). ROC curves are shown in supplementary Fig. 1c.

As with the anti-LKM1 analysis, we performed a comparison between TP, FN, FP, and TN with pIgG levels using the in-house anti-SLA iELISA as standard. FP samples from the three commercial kits showed a trend towards higher pIgG levels in FP samples, however without reaching statistical significance, possibly consequent to small numbers of FP samples (Fig. 3b).

In summary, none of the commercial anti-SLA ELISAs showed high agreement with the in-house anti-SLA iELISA, all at the expense of FN results. Alternative cut-off value did not significantly improve the agreement of the commercial assays with the iELISA. PIgG levels show a non-significant trend in FP samples measured by the commercial ELISAS.

## Discussion

Although IFT is the reference technique for the detection of AMA and anti-LKM, its methodology entails a variety of challenges. Anti-LKM1 may be easily misdiagnosed as AMA^5^, especially if the stomach substrate, to which anti-LKM1 do not bind, is not used. Therefore, highly trained and experienced personnel are required to correctly interpret the patterns; hence, there is a risk of inter-observer variability. In addition, the procedure is not automated and therefore time consuming^3,19^. Similarly, iELISA for the detection of anti-SLA is also a complex and time consuming technique. The difficulty in performing such techniques, together with the identification of specific antigenic targets for each antibody, has led to the widespread use of commercial ELISAs as a practical and affordable alternative for the detection of AMA, anti-LKM1, and anti-SLA, especially in non-specialized centers where the diagnostic evaluation of most patients with suspected AILD usually begins. External validation of these commercial assays is essential to avoid misdiagnosis and misclassification in the evaluation of patients with suspected AILD. In addition, the recently reported phenomenon of pIgG, especially in patients with untreated AIH, could lead to cross-reactivity and thus FP results and misclassification of these patients^8^.

In the case of AMA, the agreement between AMA commercial ELISAs, IFT and the in-house iELISA showed high variability. The Inova Diagnostics ELISA could replace AMA IFT and AMA iELISA in routine AMA diagnostic because it has high agreement for AMA detection. On the other hand, Aeskulisa had low agreement. Therefore, the probability of FN results is too high to use it as a screening tool in the work-up of potential PBC. The computation of an alternative and local cut-off helped to increase the agreement of the Euroimmun assay from moderate to high. However, this improvement was not observed for the Aeskulisa assay. Discrepancies between commercial ELISAs and IFT have been previously reported for AMA detection^20,21^. Previous reports of agreement between IFT and commercial AMA ELISAs have described moderate or good agreement, also at the expense of a lower sensitivity^21^. The three assays use different antigens for the detection of AMA. Aeskulisa uses a native M2 antigen, whereas Inova Diagnostics uses a purified recombinant antigen (MIT3) and Euroimmun uses a mixture of native (Bovine heart) and recombinant antigens. Assays using recombinant antigens (Inova Diagnostics and Euroimmun) appear to have a better sensitivity compared to the assay using native antigen (Aeskulisa), using the IFT and in-house iELISA as a reference, which has also been previously reported^21^. In theory, an assay using native antigens should closely match the characteristics of antigens in clinical samples. However, their standardization is more difficult than that of recombinant antigens. Our results indicate, however, that recombinant technology seems to mimic the AMA native antigens present in the IFT and used for the iELISA better. Antigen conformation has shown to play a pivotal role in the detection of specific epitopes in the accuracy of solid-phase ELISAs per se^22^. With the antigenic properties of commercial tests only being known by the respective manufacturers, we cannot clarify the exact mechanism leading to the results we observed.

For anti-LKM1, all commercial tests showed moderate or inadequate agreement with anti-LKM assessed by IFT. The correlation improved with respect to iELISA, but only Euroimmun showed a high correlation. Anti-LKM1 has a clearly identified target, namely CYP2D6^19^. In contrast to AMA, the three commercial assays evaluated use recombinant p450 IID6, which might explain a more homogeneous agreement between the commercial assays and the reference methods. Contrary to our results, a previous report showed a moderate or even high correlation between commercial anti-LKM1 ELISAs, which also use recombinant antigens, and IFT^7^. However, anti-LKM positivity on IFT is not only anti-LKM1 but might also be anti-LKM2 or any unspecified anti-LKM antibodies not belonging to classes 1–3, e.g. such as those seen in some drug- or vaccine-induced liver injury^23^. Although anti-LKM3 has been detected in 17% of AIH type 2 patients, it needs to be evaluated on human or primate substrate^24,25^. Hence, the difference in our study is not explainable by positivity for anti-LKM3 but more likely because of non-specified anti-LKM antibodies that are detected by IFT but are not anti-LKM1 as validated by ELISA. Overall, data is scarce on the role of non-anti-LKM1 anti-LKM antibodies in AIH and only assays to detect anti-LKM1 are commercially available, limiting the detection of other subtypes to selected specialized laboratories. The agreement of commercial assays with iELISA for the detection of anti-LKM1 was high (Euroimmun), moderate (Inova Diagnostics) or inadequate (Aeskulisa). This discrepancy highlights the importance of adhering to EASL guidelines in terms of using IFT for screening, as it detects not only anti-LKM1 antibodies but also every subtype, including anti-LKM3 if sera are additionally tested on human or primate tissue^24,25^. For the time being, and based on our results, commercial ELISAs cannot replace IFT for the screening for anti-LKM but being certified could safely replace self-made iELISAs for the confirmation of anti-LKM1 presence in patients with anti-LKM positivity on IFT.

For anti-SLA, none of the commercial assays showed a high correlation with the standard test, iELISA. Also, for anti-SLA there is a clearly identified antigen, allowing the development of commercial ELISAs^26^. All three anti-SLA commercial ELISAs are based on recombinant antigens. However, the high rate of FN in molecular-based assays could be explained by the fact that these assays only identify antibodies that react with linear epitopes, as they contain antigens expressed by prokaryotes and not by eukaryotic cells^5,27^. In the case of the in-house iELISA SLA antigens were derived from rat liver thereby containing their natural conformation as they are not plate-bound in the iELISA, contrary to commercial assays, but attach to naturally occurring IgGs directed against SLA, finally keeping their natural conformation and antigenic properties^4,14^.

Overall, commercial assays, especially for anti-LKM1 and anti-SLA, lack agreement compared to the respective gold standard for antibody detection recommended by current EASL guideline for AILD^2^. For anti-extractable nuclear antigen antibodies, it has been shown that significant differences in sensitivity and specificity are observed depending on the methodology^28^. For native antigen formulations, overly rigorous purification can lead to denaturation of the protein, resulting in loss of conformational epitopes relevant for antibody detection. In the case of recombinant antigens, which are usually expressed in Escherichia coli, antigenic formulations may be contaminated with E. coli protein that are falsely detected by patients’ IgG, leading to FP results or may lack post-translational modification from Eukaryotes^13^. In addition, protein synthesis in E. coli might be flawed in terms of generation of the necessary secondary and tertiary protein structure thereby giving rise to FN results.

Recently, we have demonstrated the phenomenon of pIgG in AIH patients to be a promising tool for the diagnosis of AIH^8^. PIgG binds a variety of protein and non-protein agents typically used as blocking reagents in ELISAs, potentially leading to FP test results. In this report, the number of FPs was low, therefore, although a trend towards higher pIgG levels in FP samples was observed, no significant difference was reached. However, even if this was the case, elevated pIgG levels are an accurate marker of untreated AIH^8^. Therefore, this would not imply a risk of misdiagnosis, but rather a risk of misclassification in the case of anti-LKM1 and an inaccurate evaluation of the prognosis of the patients, since anti-SLA-positive patients have a higher risk of relapse after immunosuppression withdrawal and hence often require lifelong immunosuppression^29^. Patients with high pIgG levels should undergo serologic reassessment after successful treatment, as pIgG decline within weeks to months on immunosuppressive therapy. Reassessment should be performed in expert centers or associated expert laboratories, providing the full methodological spectrum of autoantibody testing, especially in patients diagnosed with commercial ELISAs. Further evaluation of pIgG as a confounder in immunoassays should be carried out in larger multicentric cohorts to achieve enough statistical power.

This study has obvious limitations. Anti-LKM1 and anti-SLA are rare antibodies in a rare disease, which prevents the availability of larger cohorts, especially in a single-center study where the cohort was simply limited to all available antibody-positive samples. Selection by antibody negativity for the control group could be a source of bias, however, it is important to emphasize that this study was designed to evaluate the agreement of commercial ELISAs with the recommended reference gold standard techniques for antibody detection and not the accuracy of these assays for the diagnosis of a specific disease, which would require another study design.

In conclusion, this study shows that commercial ELISAs cannot completely replace the reference methods IFT and iELISA for the diagnosis of AILD, but can at least partially replace iELISA in a second confirmatory step after IFT in the diagnostic algorithm. This is consistent with current EASL guidelines, which do not recommend commercial ELISA techniques as the sole screening method for the diagnosis of AILD because of their often lower sensitivity. Not all commercial assays in our study had high agreement with the gold standard techniques, depending on the design and calibration of the assay by the manufacturer but local optimization of the cut-off only partially alleviated this problem. Hence, if there is a clinical suspicion of an AILD, patients should be referred to a center with the capacity of performing reference techniques for antibody testing. These results highlight the relevance of external validation of commercial assays, which are rarely performed, despite their clinical relevance.

## Supplementary Information

Supplementary Information.

## Data Availability

The data of this study are available from the corresponding author upon reasonable request.
