# Supplementary material for: Evaluation of immunoserological detection of anti-liver kidney microsomal, anti-soluble liver antigen and anti-mitochondrial antibodies
Source: Sci Rep. 2023 Jun 20;13:10038. doi: 10.1038/s41598-023-37095-z (PMC10282044; doi:10.1038/s41598-023-37095-z)
Supplement: Supplementary file 1 — Supplementary Information. [file 41598_2023_37095_MOESM1_ESM.pdf]

Supplementary Data

Evaluation of immunoserological quantification of anti-liver kidney microsomal, anti-soluble liver antigen and anti-mitochondrial antibodies

Alejandro Campos-Murguia[1, 2], Nicole Henjes[1, 2], Stephanie Loges[1, 2], Heiner Wedemeyer[1, 2], Elmar Jaeckel[1, 2, #], Richard Taubert[1, 2], Bastian Engel[1, 2]

Supplementary Material and Methods:

Measurement of antibodies by in-house competitive iELISA assays

Antibodies from defined anti-SLA/anti-LKM1 or AMA-positive indicator sera were coated to microtiter plates overnight at room temperature at a concentration of 20µg/ml (Dynatech, el Paso, Texas for anti-LKM1, and Maxisorp, Nunc, Denmark for AMA and SLA). Supernatants were removed and plates were washed once with phosphate buffered saline (PBS) (Sigma, ref: D5652-10L) containing 0.1% Tween 20 (Sigma, ref: P1379). Respective antigens (mitochondrial fraction for the detection of pyruvate dehydrogenase (PDH)/ branched-chain alpha-ketoacid dehydrogenase complex (BCKD), soluble fraction of 100.000x g^{-1} centrifuged rat liver for anti-SLA or microsomes from rat liver for anti-LKM1) were added at a concentration of 100µg/ml and incubated for one hour at room temperature. For the generation of the antigens rat liver was homogenized and centrifuged for 15 minutes at 3000 rpm. The pellet was discarded and the supernatant was centrifuged at 8500 rpm for 15 minutes. The mitochondrial fraction was collected from the pellet and used as antigen in the respective iELISA. The supernatant was further centrifuged for 60 min at 50000 rpm, antigens for LKM are collected from the pellet and antigens for SLA are collected from the supernatant and were added to the respective iELISA. After washing twice, patient sera, diluted 1:10 in 10mM ethylenedinitrilotetraacetic acid (Merck, ref:1.08418.0250), were added to the plates and incubated overnight at room temperature. After washing three times, biotin (Sigma, ref: B2643)-conjugated anti-IgG antibodies to respective antigens (diluted 1:100 (v/v) in PBS + 1 % bovine serum albumin (BSA) (Serva, ref: 11924.03)) were added and incubated for one hour at room temperature. After three additional wash steps, avidin-peroxidase (Dianova/Biozol, ref: 016-030-084) was added at a dilution of 1:250 (v/v) in PBS and 1% BSA and incubated for one hour at room temperature. ABTS substrate (Serva, ref: 14364.01) and sodium perborate (Merck, ref: 1.06560.100) were dissolved in citrate buffer and added to microtiter plates. The photometric reaction was stopped after five minutes with citric

acid buffer (Merck, ref: 1.00244.0500) and the absorbance was read at 405nm. The percentage of inhibition of the indicator serum to its respective autoantigen was used as a surrogate for the antibody titer.

Supplementary Figure 1.

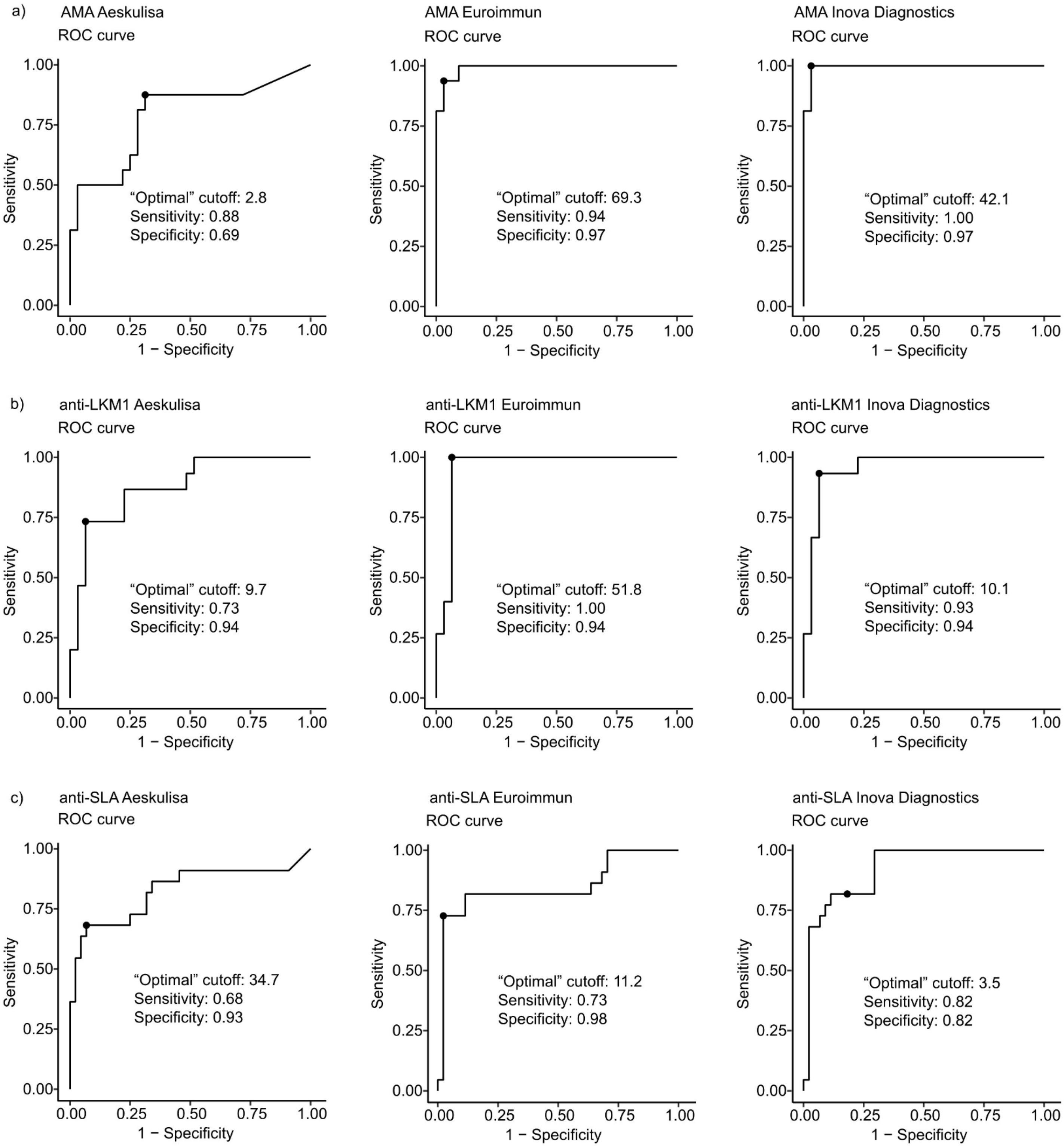

Supplementary Figure 1. Receiver operating characteristic (ROC) curve of commercial autoantibodies for detection of autoantibodies. “Optimal” cutoff based on the maximal Youden index. The sensitivity and specificity are based on the presence of autoantibodies by the reference techniques (IFT or iELISA) and not on the final disease diagnosis. a) ROC curves of commercial ELISA for the detection of AMA based on IFT. b) ROC curves of commercial ELISA for the detection of anti-LKM1 based on iELISA, since IFT can also detect other non-anti-LKM1 antibodies. c) ROC curves of commercial ELISA for the detection of anti-SLA based on iELISA.
